# Utilizing genomics and historical data to optimize gene pools for new breeding programs: A case study in winter wheat

**DOI:** 10.3389/fgene.2022.964684

**Published:** 2022-10-07

**Authors:** Carolina Ballén-Taborda, Jeanette Lyerly, Jared Smith, Kimberly Howell, Gina Brown-Guedira, Md. Ali Babar, Stephen A. Harrison, Richard E. Mason, Mohamed Mergoum, J. Paul Murphy, Russell Sutton, Carl A. Griffey, Richard E. Boyles

**Affiliations:** ^1^ Department of Plant and Environmental Sciences, Clemson University, Clemson, SC, United States; ^2^ Pee Dee Research and Education Center, Clemson University, Florence, SC, United States; ^3^ Crop and Soil Sciences Department, North Carolina State University, Raleigh, NC, United States; ^4^ U.S. Department of Agriculture-Agricultural Research Service (USDA-ARS), Raleigh, NC, United States; ^5^ Agronomy Department, University of Florida, Gainesville, FL, United States; ^6^ School of Plant, Environmental and Soil Sciences, Louisiana State University, Baton Rouge, LA, United States; ^7^ College of Agricultural Sciences, Colorado State University, Fort Collins, CO, United States; ^8^ Department of Crop and Soil Sciences, University of Georgia, Griffin, GA, United States; ^9^ Department of Soil and Crop Sciences, Texas A&M University, Commerce, TX, United States; ^10^ School of Plant and Environmental Sciences, Virginia Tech, Blacksburg, VA, United States

**Keywords:** breeding, winter wheat (*Triticum aestivum* L.), historical data, training populations, genomic selection, prediction accuracy, yield

## Abstract

With the rapid generation and preservation of both genomic and phenotypic information for many genotypes within crops and across locations, emerging breeding programs have a valuable opportunity to leverage these resources to 1) establish the most appropriate genetic foundation at program inception and 2) implement robust genomic prediction platforms that can effectively select future breeding lines. Integrating genomics-enabled^1^ breeding into cultivar development can save costs and allow resources to be reallocated towards advanced (i.e., later) stages of field evaluation, which can facilitate an increased number of testing locations and replicates within locations. In this context, a reestablished winter wheat breeding program was used as a case study to understand best practices to leverage and tailor existing genomic and phenotypic resources to determine optimal genetics for a specific target population of environments. First, historical multi-environment phenotype data, representing 1,285 advanced breeding lines, were compiled from multi-institutional testing as part of the SunGrains cooperative and used to produce GGE biplots and PCA for yield. Locations were clustered based on highly correlated line performance among the target population of environments into 22 subsets. For each of the subsets generated, EMMs and BLUPs were calculated using linear models with the *‘lme4’* R package. Second, for each subset, TPs representative of the new SC breeding lines were determined based on genetic relatedness using the *‘STPGA’* R package. Third, for each TP, phenotypic values and SNP data were incorporated into the *‘rrBLUP’* mixed models for generation of GEBVs of YLD, TW, HD and PH. Using a five-fold cross-validation strategy, an average accuracy of *r* = 0.42 was obtained for yield between all TPs. The validation performed with 58 SC elite breeding lines resulted in an accuracy of *r* = 0.62 when the TP included complete historical data. Lastly, QTL-by-environment interaction for 18 major effect genes across three geographic regions was examined. Lines harboring major QTL in the absence of disease could potentially underperform (e.g., Fhb1 R-gene), whereas it is advantageous to express a major QTL under biotic pressure (e.g., stripe rust R-gene). This study highlights the importance of genomics-enabled breeding and multi-institutional partnerships to accelerate cultivar development.

## 1 Introduction

Wheat (*Triticum aestivum* L.) is a major cereal crop worldwide as its production ranks third in the US (49.7 million tonnes) and globally (895.2 million tonnes) behind maize and soybean. Wheat has a high production value of US$8.7 billion in the United States and $188.1 billion globally (FAOSTAT 2022). The effects of climate change, including warming temperatures, variable precipitation and more frequent extreme weather events (Simpson and Burpee 2014), as well as diseases (Singh et al., 2016), are challenging wheat yield potential and causing increased yield instability across years (Hatfield and Dold 2018). Development of resilient, high-yielding wheat cultivars with stable grain production across target population of environments is essential (Braun et al., 1992; Langridge and Reynolds 2021). Multi-environment trials in major production areas facilitate yield potential and stability assessment of advanced breeding lines and provide information to identify and understand complex genotype-by-environment interactions (GE) (Dwivedi et al., 2020). However, collecting data in multiple locations and years for many early-stage breeding lines has high labor and economic costs, which imposes a need to integrate genomics-enabled breeding (e.g., genomic selection) and data-driven methods to accelerate the breeding process (Rincent et al., 2017; Juliana et al., 2020). Establishing an alliance of breeding programs that share target environments is crucial for data sharing, germplasm exchange, and for conducting advanced regional trials of candidates for release (Chenu 2015; Spindel and McCouch 2016; Sarinelli et al., 2019).

Genomic selection (GS) is becoming a valuable technology for modern crop breeding programs, and its implementation for cultivar development has been shown to accelerate the rate of genetic gain by shortening the breeding cycle and/or increasing selection accuracy (Crossa et al., 2017; Voss-Fels et al., 2019). Genomic selection uses established genotype and phenotype data of a training population (TP) to calibrate a prediction model, which is then used to estimate trait genomics breeding values (GEBVs) of untested new genotypes. Based on GEBVs, superior breeding lines are selected at preliminary stages prior to phenotyping (Voss-Fels et al., 2019). Earlier selection allows breeders increase breeding efficiency and save costs (Crossa et al., 2017) by reducing the number of promising breeding lines that need to be evaluated in advanced multi-environment and replicated field trials (Wartha and Lorenz 2021).

There is increased interest in incorporating historical datasets into genomic prediction models (Dawson et al., 2013). Here, historical data refers to preexisting data collected by breeding programs over time that were not generated specifically for genomic selection modeling. Using historical datasets could be beneficial for GS if the target population of environments have been accurately evaluated within advanced trials over time, the dataset is large, and the focal trait possesses high heritability (Rutkoski et al., 2015). Several studies have incorporated historical data into genomic prediction models to predict economically important traits including grain yield (YLD) in wheat, with reports of moderate-to-high accuracies in local breeding programs of *r* = 0.50 in France (Storlie and Charmet 2013) and *r* = 0.64 in the US (Sarinelli et al., 2019), as well as accuracies of *r* = 0.85 in an international cultivar development program (Dawson et al., 2013). GS has been used to enhance the primary target trait YLD, but it is also useful to predict and select other important traits such as disease resistance, including stem rust resistance (Rutkoski et al., 2015) and *Fusarium* head blight resistance (FHB) (Rutkoski et al., 2012), agronomic traits such as test weight (TW), heading date (HD) and plant height (PH) (Gill et al., 2021) and quality-related traits including protein content, starch content, and flour yield (Tsai et al., 2020; Sandhu et al., 2022). Lastly, GS can be applied for selection of low-heritable complex traits that are expensive or difficult to measure, or by including high-heritable correlated secondary traits into models (Rutkoski et al., 2016; Sapkota et al., 2020).

This study was conducted to understand how new or reestablished breeding programs should leverage existing historical genomic, and multi-environment and multi-trait phenotype data of elite breeding lines. The Clemson University winter wheat breeding program was reestablished in 2017 and used as a case study to understand the foundational genetics and requirements to maximize predictive ability of genomic models to successfully develop cultivars adapted to a target population of environments. To accomplish this, historical genotypic and phenotypic information for advanced soft red winter (SRW) wheat breeding lines, evaluated as part of the Southeastern University small grains (SunGrains) breeding alliance, was used to predict YLD, TW, HD and PH using optimized TPs for a set of untested SC-derived breeding lines. Two validation strategies were completed to assess and compare fitted models’ prediction accuracy. Finally, QTL-by-environment (QE) interaction analysis was completed using 18 major effect QTL to identify whether there was a favorable effect on yield across three major testing regions. The use of comprehensive datasets and genomic models have great value to securing the needed increases in genetic gain and enhance the efficiency of cultivar development.

## 2 Materials and methods

### 2.1 Plant materials

**FIGURE 1:**
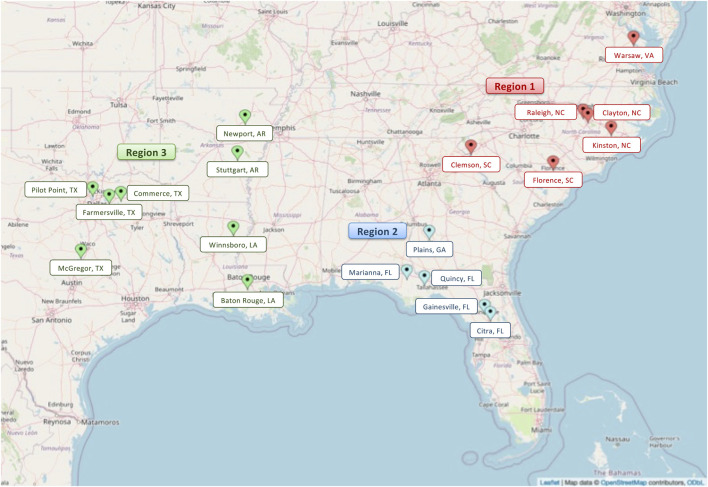
Map indicating the three major target population of environments (regions) (Boyles et al., 2019). In red indicating the Atlantic coastal plain correlated trial locations (region 1), in blue Georgia and Florida locations (region 2) and in green the gulf coast locations (region 3). Pinpointed are the 19 locations in eight states of the Southeastern US region were SunGrains breeding lines are evaluated annually.

Annually, advanced SRW wheat lines entered into the Gulf Atlantic wheat nursery (GAWN) and advanced wheat nursery (SunWheat) are evaluated across the greater southeastern US, which is coordinated by SunGrains and partnering public wheat breeding programs. The SunGrains cooperative includes seven land-grant university breeding programs (Clemson University, NC State University, Louisiana State University, Texas A&M University, University of Arkansas, University of Florida, and the University of Georgia) having very strong collaborations for field evaluation and unfettered distribution of adapted germplasm and data exchange. As part of this historical cooperative, a total of 1,285 lines were tested in 19 locations (Figure 1 and Supplementary Table S1) from 2008 to 2021. On average, 108 breeding lines (ranging from 56 in 2011 and 161 in 2021) along with several commercial checks were evaluated annually in field trials. The MapCustomizer^2^ web plotting tool was used to generate a map with trial locations. Data from 2008 to 2020 was used for GS analysis, and data from 2008 to 2021 was used for QE analysis.

### 2.2 Historical phenotype data

Historical phenotypic data consisted of a multi-location, multi-year and multi-trait dataset generated and maintained by SunGrains. A total of 1,285 elite SRW wheat breeding lines were tested in two regional nurseries (GAWN and SunWheat) in 19 trial locations in the southeastern US (Figure 1 and Supplementary Table S1). The number of observations for YLD (kg ha^−1^), TW (kg hl^−1^), HD (Julian days) and PH (cm) was 17,645, 14,942, 11,092 and 8,678, respectively. The number of replications ranged from one to three, depending on location-year combination.

Two analyses were performed to determine appropriate subsets of location-year combinations to include in the phenotypic dataset for optimizing the GS model for the target population of environments. First, the historical phenotypic dataset was used to display principal component (PCA) plots from Pearson’s correlation matrix using the *‘princomp’*, *‘cor’* and *‘corrplot’* packages in R; second, biplots showing the relationship among environments (Yan et al., 2000) were obtained using a genotype plus genotype-by-environment (GGE) model using the *‘gge’* (Wright and Laffont 2018) and *‘GGEBiplots’* (Dumble et al., 2017) packages in R. This analysis was repeated for the GAWN, SunWheat, and combined (GAWN + SunWheat) phenotypic datasets to select eight, four and ten subgroups of trial locations, respectively (Supplementary Table S1).

For each of the subsets of locations (except for one group that had low number of datapoints for estimation of genetic values), the following linear model (Yao et al., 2018) was fitted using the function *‘lmer’* of the *‘lme4’* package in R (Bates et al., 2015) to estimate genetic values for YLD, TW, HD, and PH:

$$Y_{\mathrm{ijk}}{\;=µ+G}_{i}{\;+E}_{j}{\;+R}_{k\left(j\right)}{\;+\mathrm{GE}}_{\mathrm{ij}}{\;+e}_{\mathrm{ijk}}$$

Where *Y*_*ijk*_ represents the phenotypic observation of genotype *i* in environment *j* and replication *k*, *µ* is the overall mean; *G*_*i*_ is the effect of genotype *i*, *E*_*j*_ is the effect of environment (location-year combination) *j*; *R*_*k(j)*_ the effect of replication *k* nested in environment *j*; *GE*_*ij*_ the G × E interaction between genotype *i* and environment *j*; and *e*_*ijk*_ the residual effect associated with genotype *i* in environment *j* and replication *k*. All terms except genotype were set as random effects. Genotype was defined as fixed effect (Lado et al., 2017) to estimate marginal means (EMMs) using the *‘emmeans’* package in R, and as random effect (Yao et al., 2018) to calculate best linear unbiased predictors (BLUPs) using the *‘coef’* and *‘ranef’* functions of the *‘lme4’* package in R. Pearson’s correlations between EMMs and BLUPs were analyzed with the *‘corrplot’* packages in R (Wei and Simko 2017). Variance components of the linear models fitted with the *‘lme4’* package were used to estimate broad-sense heritability (H^2^) with the *‘H2cal’* function of the *‘inti’* R package (Lozano-Isla 2022). The Cullis method (Cullis et al., 2006), recommended for unbalanced, multi-environment datasets (Covarrubias-Pazaran 2019), was used according to the following equation where genotype was a random effect:

$$H_{Cullis}^{2}=1-\frac{{\bar{V}}_{\Delta }^{BLUP}}{2*\sigma_{G}^{2}}$$

where ${\bar{V}}_{\Delta }^{BLUP}$ is the mean variance of genotypic BLUPs and $\sigma_{G}^{2}\;$ represents the genetic variance ( ΔG , genetic gain).

### 2.3 Genotype data

Genotyping was performed similarly to methods previously described (Sarinelli et al., 2019; Winn et al., 2022). DNA was extracted using sbeadex plant maxi kits (LGC Genomics, Middlesex, United Kingdom) according to the manufacturer’s instructions. Genotyping-by-sequencing (GBS) was performed as previously described (Poland JA. et al., 2012) and libraries were sequenced on an Illumina HiSeq 2500 or NovaSeq 6000 at the USDA-ARS Eastern Regional Small Grains Genotyping laboratory (Raleigh, NC). Reads were mapped to the wheat genome assembly (RefSeq 1.0) (Appels et al., 2018) using the Burrows-Wheeler aligner (BWA) (v.0.7.12) (Li and Durbin 2009) and single nucleotide polymorphism (SNP) discovery was completed with Tassel-5GBSv2 (v.5.2.35) (Glaubitz et al., 2014). Data was filtered by removing taxa with >85% missing data, while retaining SNPs with ≥5% minor allele frequency (MAF), ≤10% of heterozygous proportion and missing data of ≤20%. Finally, missing data was imputed with Beagle v5.1 (Browning and Browning 2007; Browning et al., 2018). Exported VCF file containing 1,149 elite lines tested in advanced trials from 2008 to 2020 and the 1,133 breeding lines from SC sequenced in 2020 and 2021 was filtered. SNPs with MAF of less than 5% were discarded and a maximum heterozygous proportion of 10% was allowed (Juliana et al., 2020). A total of 15,077 SNPs for 9,137 genotypes were exported as a HapMap file and converted into a numerical matrix (0,1,2) using GAPIT (v.3.1.0) with default parameters in R (Lipka et al., 2012).

### 2.4 Training population selection

Training population optimization was performed to target strategic production environments within the southeastern US. A total of 998 (361 from 2020 to 637 from 2021) new SC breeding lines were used to identify the best TP using each of the subsets of trial locations (Supplementary Table S1). The best TPs of 400 individuals were selected from the 1,149 SunGrains breeding lines based on the genetic relatedness (Norman et al., 2018) to the 998 lines in the prediction set. The R *‘STPGA’* package (Akdemir et al., 2015; Akdemir 2017) was utilized using the historical high-density genotype dataset with the following parameters: the genetic algorithm was *GenAlgForSubsetSelection’*, the optimality criteria was *‘PEVmean’*, ‘*nelite’* was set to 10, population size was set to 400 (Isidro et al., 2015; Michel et al., 2017; Sarinelli et al., 2019), and other parameters were set with default values (Sarinelli et al., 2019). The first 100 principal components calculated from the genotype data were chosen for prediction of error variance. Optimal TPs were selected after 300 iterations and 10 replications.

The frequency and percentage (%) of breeding lines by breeding program selected by STPGA was calculated for each TP and normalized by number of lines by program. A stacked barplot was displayed with *‘ggplot’* package in R (Wickham 2016). A heatmap was obtained with ‘*pheatmap’* package (Kolde 2012), and PCAs using genotypic data were calculated with ‘*prcomp*’ package and plotted with *‘ggplot’*.

### 2.5 Genomic selection and cross-validation

Genomic best linear unbiased prediction (GBLUP) mixed models were fitted to estimate GEBVs for YLD, TW, TW and PH with the following equation:

y = Xβ + Zμ + e

Where **y** represents the vector of BLUEs for each genotype; **X** and **Z** represent the design matrices for fixed and random effects, respectively; **β** is the vector of fixed effects; **μ** is the vector for random genotypic effects; and **e** is a vector of residuals (Sarinelli et al., 2019). EMMs and BLUPs of each of the 22 TPs selected by STPGA (Supplementary Table S2) and the SNP dataset were entered into the ‘*mixed.solve*’ function of the R ‘*rrBLUP*’ package (Endelman 2011) for marker-based predictions. The restricted maximum-likelihood method (REML) was used, and other parameters were set as default.

Two types of validation were implemented to assess and compare each model’s prediction ability using each TP for each trait. First, five-fold cross-validation (CV) is a procedure that randomly divides the TP into five groups of approximately equal size (20%). One random group is masked and GEBVs are calculated for the masked set using the remaining four folds (80% of lines) (Lozada and Carter 2019). After completing this step for all five folds, the correlations between observed values (EMMs/BLUPs) and the predicted values (GEBVs) were used to assess the accuracy of prediction by averaging the five correlations. Second, a validation was performed using data from 58 advanced breeding lines that were selected and developed in SC for regional testing in 2021; six lines tested in GAWN, 14 in SunWheat, 19 in SunGrains preliminary early (SPE) nursery and 19 in SunGrains preliminary late (SPL) nursery. For GAWN and SunWheat nurseries, YLD was measured in nine locations (Warsaw, VA; Kinston, NC; Clemson, SC; Florence, SC; Plains, GA; Citra, FL; Marianna, AR; McGregor, TX, Winnsboro, LA.). SPE and SPL included phenotypic data from seven locations (Marianna, AR; Plains, GA; Gainesville, FL; Winnsboro, LA; Kinston, NC; Florence, SC; and McGregor, TX.). EMMs and BLUPs were calculated for data collected in all locations, as well as data collected exclusively in Florence, SC and its most similar trial location Kinston, NC (Boyles et al., 2019). These two datasets were used for comparison with GEBVs calculated with the 22 TPs selected by STPGA. Four-quadrant plots showing the correlation between observed and predicted values were displayed with the *‘ggplot’* package in R. Means of observed and predicted values were calculated to divide the plot into four quadrants: A (upper-right) and B (lower-left) for correctly classified lines, and C (lower-right) and D (upper-left) for wrongly categorized genotypes.

Furthermore, GBLUP mixed models were fitted to estimate GEBVs for the 998 breeding lines developed by the Clemson University breeding program. Using the EMMs and BLUPs of the TP with highest prediction accuracy, predictions of YLD, TW, HD and PH were performed with the *‘mixed.solve’* function of the R ‘*rrBLUP’* package as previously described. With the aim to select a subset of lines as the foundation for the breeding program as well as first year advanced field testing, one hundred and five superior lines were identified based on GEBVs for YLD, and 20 low-ranked lines were identified for comparison. Advanced field evaluation for these lines is in progress (data not shown).

### 2.6 QTL-by-environment

SunGrains’ elite breeding lines have been evaluated every year with Kompetitive allele-specific PCR assays (KASP, LGC Biosearch Technologies, Hoddesdon, United Kingdom) to generate composite calls for major effect genes at the Eastern Regional Genotyping Small Grains Laboratory (Raleigh, NC). Over the course of 14 years (2008–2021), 4,426 breeding lines were tested for 75 molecular markers associated with disease/pest resistance, photoperiod, vernalization, dwarfing, grain texture and kernel color (Díaz et al., 2012; Guedira et al., 2016; Mason et al., 2018; Sarinelli et al., 2019). For this study, 18 of the 75 major genes were selected due to a given association with one or more of the following: FHB (*Fusarium graminearum*) resistance, leaf rust (*Puccinia triticina*) resistance, stem rust (*P. graminis*) resistance, stripe rust (*P. striiformis*) resistance, Hessian fly (*Mayetiola destructor*) resistance, powdery mildew (*Blumeria graminis*) resistance, septoria nodorum blotch (*Parastagonospora nodorum*) susceptibility, or photoperiod sensitivity (Supplementary Table S3). In addition to their association with important traits, these genes were also selected because of their high frequency among regional, SRW wheat lines and their perceived value to resiliency and productivity.

Historical phenotype data from 1,285 breeding lines was used to assess the effect on agronomic traits when the expression of a major effect QTL differs under different environmental pressure (e.g., low or high pest/disease pressure) across production locations (Lowry et al., 2019). The historical dataset was compartmentalized into two different ways: 1) target population of environments (Boyles et al., 2019), which was a set of three mega-environments herein referred to as regions based on testing location; and 2) breeding line origin which considered potential genetic background bias. Region 1 included all data collected in states located in the Atlantic Coastal Plain (NC, SC, VA), Region 2 comprised data from GA and FL locations, and Region 3 represented data from Gulf Coast states (TX, AR, LA) (Figure 1 and Supplementary Table S4). Breeding line origin was categorized using the same three groups. A total of 1,172 lines shared between this historical phenotypic dataset and the Eastern Regional Marker Report were selected for QE analysis.

For each of the 18 major genes, only absent/present calls were considered for analysis by discarding heterozygous, null, and failed calls. The following linear mixed model was calculated to test the significance of QTL-by-environment (region/origin) interactions for YLD, TW and HD using the function *‘lmer’* of *‘lme4’* package in R:

$$Y_{\mathrm{ijk}}{\;=\mu +G}_{i}{\;+E}_{j}{\;+R}_{k\left(j\right)}{\;+\mathrm{GE}}_{\mathrm{ij}}{+\mathrm{QE}+e}_{\mathrm{ijk}}$$

Where *Y*_*ijk*_ represents the phenotypic observation of genotype *i* in environment *j* and replication *k*, *µ* the overall mean; *G*_*i*_ is the effect of genotype *i*, *E*_*j*_ is the effect of environment (location-year combination) *j*; *R*_*k(j)*_ the effect of replication *k* nested in environment *j*; *GE*_*ij*_ the G x E interaction between genotype *i* and environment *j*; *QE* the QTL-by-environment interaction effect; and *e*_*ijk*_ the residual effect associated with genotype *i* in environment *j* and replication *k*. QE effect was considered fixed and all the remaining effects were considered random.

Using the mixed model, EMMs were calculated using the *‘emmeans’* function in R for the fixed effect of the interaction between major gene (absent/present) and region (1, 2 and 3). To estimate the significant difference at 0.05, *p*-values were calculated using pairwise comparisons between groups with the option *‘pairwise’* and adjusted with the Tukey correction method. Plots for EMMs and *p*-values were displayed with ‘*ggplot*’ in R. The same analysis was performed for YLD using the classification of locations by origin to assess for genetic background bias.

## 3 Results

### 3.1 Summary of historical phenotype data

The historical phenotypic dataset was used to calculate biplots and PC plots showing the relationship among environments (Supplementary Figure S1). These plots allowed for the classification of locations by similarity of line performance into 22 subsets as follows: eight groups using data collected in GAWN nursery, four groups using data from SunWheat nursery and ten groups using the whole dataset. For 22 subgroups, EMMs and BLUPs were estimated in R (Supplementary Table S1). Histogram plots for the four traits using the full dataset revealed a normal distribution of EMMs and BLUPs (Supplementary Figure S2). Correlation plots exhibited strong positive relationships between predictors for each trait (Supplementary Figure S3). Because there was complete correlation (*r* = 1) between BLUPs calculated with *‘coef’* and *‘ranef’* R functions, hereinafter only results using BLUPs estimated with *‘coef’* function are presented.

Broad-sense heritability (H^2^) using the Cullis method was moderate to high for YLD (*r* ^*2*^ *=* 0.56), TW (*r* ^*2*^ *=* 0.74), HD (*r* ^*2*^ *=* 0.85) and PH (*r* ^*2*^ *=* 0.83).

### 3.2 Training population selection

**FIGURE 2:**
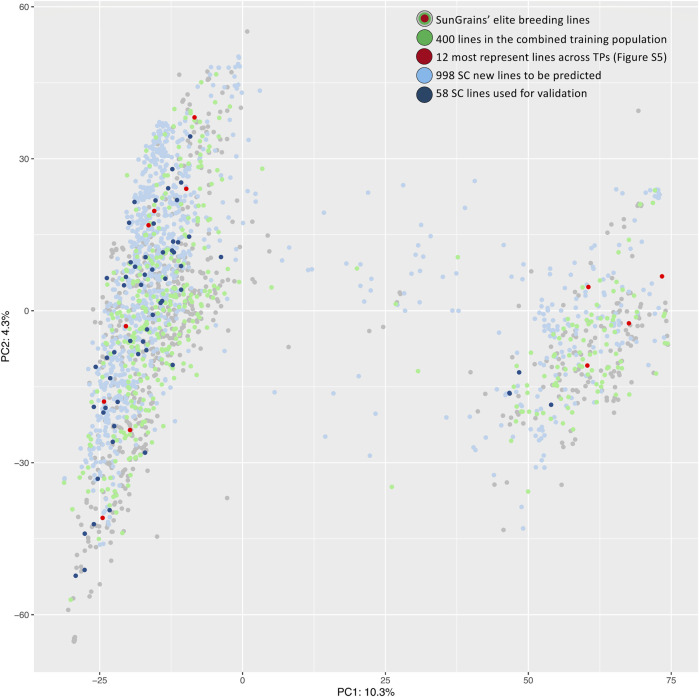
PCA plot of first two principal components is showing the genetic relationship between SunGrains’ elite lines (gray, green and red dots), the SC new breeding lines (light blue) and SC lines used for validation (dark blue). Lines selected in the combined TP (SetAll_TP) are indicated in green and lines present in at least 20 TPs (Supplementary Figure S5) are highlighted in red. Percentage represent the proportion of variance explained by each principal component.

A genotypic dataset of 15,077 SNPs and 9,137 genotypes, which included 998 SC lines and 1,149 SunGrains advanced breeding lines, was used to establish optimal TPs. Subsetting was based on genetic relatedness calculated using the *‘STPGA’* R package. TPs containing 400 SunGrains advanced breeding lines that most represented the SC prediction population were selected, with the exception of three TPs (Set11_TP and Set13_TP with 350 and Set15_TP with 300 lines) where fewer lines were selected due to a lower number of available entries. The normalized frequency of lines selected by breeding program showed that overall representation of breeding programs within TPs was LA (*µ* = 13.9%), NC (*µ* = 13.8%), AR (*µ* = 13.3%), GA (*µ* = 13.2%), TX (*µ* = 12.9%), FL (*µ* = 12.5%), VA (*µ* = 11.7%), and SC (8.8%). After normalizing the number of lines included from each program by the total lines available from each program (*n* included/*t* available * 100 = normalized %), it was apparent that STPGA included approximately one-third of individuals from every program, with a mean over all 22 sets ranging from a high of 36.3% (LA) to a low of 24% (SC) (Supplementary Figure S4 and Supplementary Table S2). The heatmap showed the lines selected by STPGA across TPs (within nursery clustering), and that 12 lines were selected in at least 20 TPs (Supplementary Figure S5). The SunGrains’ elite breeding lines and SC lines were genetically compared using PCA plots (Figure 2). STPGA selected SunGrains’ lines (Figure 2, in green) that best captured the genetics present in the new SC lines (Figure 2, in blue). The PC1 that explained 10.3% of the variation divides the genotypes into two distinct subpopulations associated with the presence/absence of the t2BS:2GS·2GL:2BL translocation derived from *T. timopheevii* (Sarinelli et al., 2019).

### 3.3 Genomic selection and cross-validation

GBLUP mixed models were fitted to predict GEBVs for YLD, TW, HD and PH using the selected 22 TPs. Using five-fold CV, a mean accuracy of *r* = 0.42 was observed across the 22 TPs when using EMMs as the observed data for YLD. When using BLUPs, the average prediction accuracy was reduced to *r* = 0.31. For TW, mean prediction accuracies ranged from *r* = 0.26 to 0.32 when using BLUPs and EMMs, respectively. Prediction accuracies oscillated from *r* = 0.42 to 0.45 for HD and PH (Table 1).

**TABLE 1:** Five-fold CV using estimated values (EMMs and BLUPs) of 22 TPs (Supplementary Table S2) and the prediction accuracy to predict GEBVs for YLD, TW, HD and PH.

Nursery	TP	YLD EMMs	YLD BLUPs	TW EMMs	TW BLUPs	HD EMMs	HD BLUPs	PH EMMs	PH BLUPs
GAWN	Set01_TP	0.53	0.42	0.34	0.33	0.46	0.45	0.54	0.53
GAWN	Set02_TP	0.44	0.35	0.30	0.26	0.37	0.35	0.43	0.45
GAWN	Set03_TP	0.55	0.45	0.36	0.28	0.51	0.53	0.43	0.37
GAWN	Set04_TP	0.47	0.37	0.30	0.24	0.45	0.43	0.46	0.45
GAWN	Set05_TP	0.47	0.41	0.36	0.31	0.34	0.37	0.42	0.44
GAWN	Set06_TP	0.46	0.36	0.37	0.33	0.33	0.33	0.38	0.39
GAWN	Set07_TP	0.47	0.42	0.34	0.27	0.40	0.43	0.45	0.47
GAWN	Set08_TP	0.46	0.35	0.37	0.30	0.37	0.40	0.52	0.53
SunWheat	Set09_TP	0.37	0.27	0.26	0.21	0.48	0.39	0.37	0.42
SunWheat	Set11_TP	0.29	0.13	0.17	0.17	0.37	0.32	0.38	0.40
SunWheat	Set12_TP	0.47	0.28	0.22	0.17	0.49	0.45	0.39	0.42
SunWheat	Set13_TP	0.29	0.20	0.23	0.17	0.41	0.30	0.48	0.47
GAWN + SunWheat	Set14_TP	0.52	0.44	0.45	0.36	0.55	0.54	0.50	0.51
GAWN + SunWheat	Set15_TP	0.16	0.15	0.03	0.05	0.45	0.45	0.34	0.35
GAWN + SunWheat	Set16_TP	0.21	0.11	0.12	0.13	0.39	0.39	0.45	0.49
GAWN + SunWheat	Set17_TP	0.36	0.21	0.42	0.28	0.38	0.44	0.40	0.44
GAWN + SunWheat	Set18_TP	0.41	0.25	0.39	0.29	0.49	0.50	0.38	0.42
GAWN + SunWheat	Set19_TP	0.49	0.34	0.39	0.27	0.43	0.46	0.44	0.45
GAWN + SunWheat	Set20_TP	0.40	0.29	0.38	0.23	0.44	0.45	0.47	0.46
GAWN + SunWheat	Set21_TP	0.50	0.38	0.40	0.30	0.45	0.46	0.43	0.43
GAWN + SunWheat	Set22_TP	0.42	0.31	0.41	0.33	0.43	0.44	0.51	0.52
GAWN + SunWheat	SetAll_TP	0.45	0.35	0.37	0.33	0.45	0.46	0.48	0.49
Average		0.42	0.31	0.32	0.26	0.43	0.42	0.44	0.45

Notes: Accuracies between 0.4–0.5 highlighted in light green and accuracies higher that 0.5 in dark green. Here presenting analysis was completed using BLUPs estimated with ‘*coef*’ function **(**BLUPs calculated with ‘*coef*’ function and ‘*ranef*’ function resulted in the same accuracies).

**FIGURE 3:**
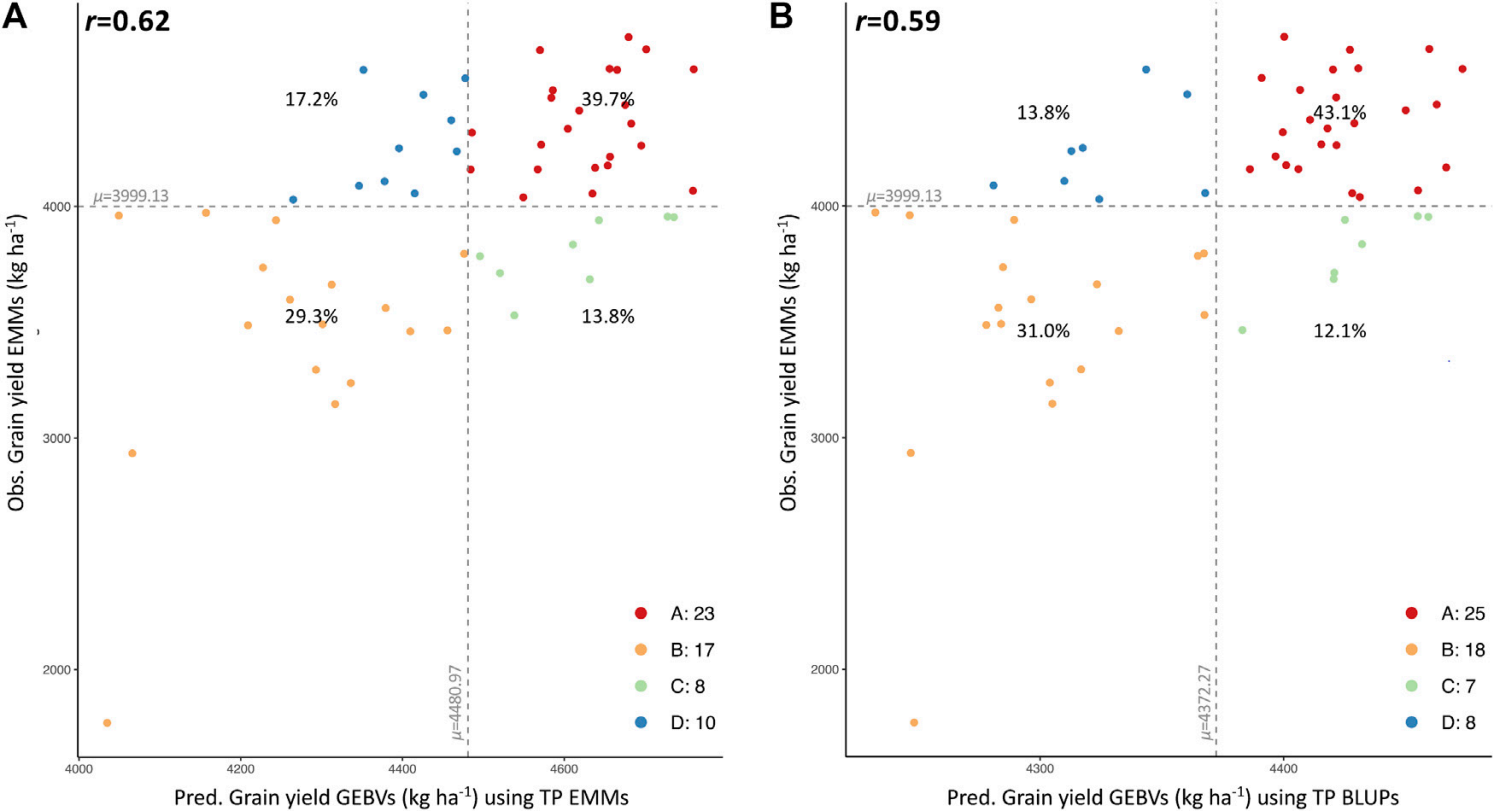
Four-quadrant plots showing the correlation between predicted (GEBVs, x-axis) and observed yield (EMMs, y-axis) for 58 SC advanced breeding lines using the combined TP data (SetAll_TP). Correlation between observed and predicted values for YLD using TP EMMs **(A)** and TP BLUPs **(B)**. Mean of observed and predicted values is dividing the plot into 4 quadrants, A (upper-right section in red), B (lower-left section yellow), C (lower-right section in green) and D (upper-left section in blue). Percentage (%) of total lines classified in each quadrant is displayed.

A smaller set of 58 new breeding lines, developed by the reestablished Clemson breeding program and evaluated for YLD in several locations in the 2020–2021 growing season, were used for additional validation. Observed and predicted values were compared to assess the predictive ability of each TP (Supplementary Table S5). Overall, YLD predictions generated from EMMs of the combined TP (historical phenotypic data-SetAll_TP) had the greatest correlation with observed data (Table 2). A prediction accuracy as high as *r* = 0.62 was obtained when comparing predicted GEBVs (predicted with the EMMs of the combined TP) and EMMs of observed YLD data. The four-quadrant plot for this comparison, where 69% (40 of 58) of breeding lines were accurately categorized into the proper quadrant (A and B) and 31% of the breeding lines were categorized in quadrants C and D (Figure 3A). A prediction accuracy of *r* = 0.59 was obtained by comparing the GEBVs (predicted with the BLUPs of the combined TP), versus the EMMs of YLD. In this case, 74.1% of the breeding lines with high or low observed YLD were categorized in quadrants A and B, whereas 25.9% of the breeding lines fell into quadrants C and D (Figure 3B). Finally, GEBVs demonstrated low correlations with observed data collected only in Florence, SC and its nearest trial in Kinston, NC (Table 2).

**TABLE 2:** Validation using estimated values (EMMs and BLUPs) of 22 TPs (Supplementary Table S2) and the prediction accuracy to predict GEBVs for YLD of 58 lines developed in Florence, SC.

Nursery	Pred. GEBVs calculated using TP (predicted values)	YLD (Obs. EMMs)	YLD (Obs. BLUPs)	YLD (Obs. EMMs SC & NC only)	YLD (Obs. BLUPs SC & NC only)
GAWN	Set01_TP (EMMs)	0.48	0.42	0.26	0.23
	Set01_TP (BLUPs)	0.41	0.34	0.26	0.19
GAWN	Set02_TP (EMMs)	0.31	0.26	0.26	0.22
	Set02_TP (BLUPs)	0.09	0.04	0.17	0.12
GAWN	Set03_TP (EMMs)	0.26	0.24	0.10	0.10
	Set03_TP (BLUPs)	0.09	0.05	−0.01	−0.04
GAWN	Set04_TP (EMMs)	0.23	0.18	0.02	−0.02
	Set04_TP (BLUPs)	0.08	0.03	−0.08	−0.12
GAWN	Set05_TP (EMMs)	0.24	0.21	0.29	0.23
	Set05_TP (BLUPs)	0.15	0.10	0.21	0.14
GAWN	Set06_TP (EMMs)	0.22	0.17	0.22	0.15
	Set06_TP (BLUPs)	0.12	0.06	0.08	0.01
GAWN	Set07_TP (EMMs)	0.27	0.22	0.21	0.15
	Set07_TP (BLUPs)	0.14	0.10	0.15	0.09
GAWN	Set08_TP (EMMs)	0.19	0.13	0.14	0.07
	Set08_TP (BLUPs)	0.06	0.01	0.05	−0.03
SunWheat	Set09_TP (EMMs)	0.48	0.43	0.16	0.11
	Set09_TP (BLUPs)	0.46	0.41	0.16	0.11
SunWheat	Set11_TP (EMMs)	0.23	0.18	−0.01	−0.01
	Set11_TP (BLUPs)	0.26	0.20	0.04	0.02
SunWheat	Set12_TP (EMMs)	0.31	0.25	0.06	−0.01
	Set12_TP (BLUPs)	0.30	0.22	0.03	−0.04
SunWheat	Set13_TP (EMMs)	0.26	0.23	0.04	0.09
	Set13_TP (BLUPs)	0.07	0.08	0.02	0.09
GAWN + SunWheat	Set14_TP (EMMs)	0.34	0.32	0.32	0.29
	Set14_TP (BLUPs)	0.37	0.33	0.26	0.23
GAWN + SunWheat	Set15_TP (EMMs)	0.52	0.53	0.37	0.39
	Set15_TP (BLUPs)	0.51	0.51	0.37	0.38
GAWN + SunWheat	Set16_TP (EMMs)	0.21	0.21	0.06	0.11
	Set16_TP (BLUPs)	0.15	0.14	0.04	0.10
GAWN + SunWheat	Set17_TP (EMMs)	0.41	0.35	0.09	0.05
	Set17_TP (BLUPs)	0.45	0.41	0.17	0.16
GAWN + SunWheat	Set18_TP (EMMs)	0.43	0.39	0.10	0.10
	Set18_TP (BLUPs)	0.41	0.37	0.13	0.14
GAWN + SunWheat	Set19_TP (EMMs)	0.45	0.41	0.15	0.13
	Set19_TP (BLUPs)	0.44	0.39	0.15	0.12
GAWN + SunWheat	Set20_TP (EMMs)	0.49	0.45	0.14	0.12
	Set20_TP (BLUPs)	0.47	0.44	0.15	0.14
GAWN + SunWheat	Set21_TP (EMMs)	0.47	0.41	0.19	0.16
	Set21_TP (BLUPs)	0.45	0.39	0.2	0.17
GAWN + SunWheat	Set22_TP (EMMs)	0.39	0.34	0.21	0.15
	Set22_TP (BLUPs)	0.37	0.32	0.25	0.18
GAWN + SunWheat	SetAll_TP (EMMs)	**0.62** (Figure 3A)	0.56	0.34	0.29
	SetAll_TP (BLUPs)	**0.59** (Figure 3B)	0.52	0.38	0.30

Notes: In parenthesis the estimated values used for predictions. Accuracies between 0.4–0.5 highlighted in light green and accuracies higher that 0.5 in dark green. In bold the higher prediction accuracies. Here presenting analysis was completed using BLUPs estimated with *‘coef’* function **(**BLUPs calculated with *‘coef’* function and *‘ranef’* function resulted in the same accuracies).

The TP that was optimized with historical data and possessed the highest prediction accuracy (*r* = 0.62) (Figure 3 and Table 2) was used for calculating GEBVs of YLD, TW, HD and PH for 998 breeding lines developed in SC (Supplementary Table S6). Based on YLD, the 105 most promising breeding lines predicted to have a superior performance (4691–5036 kg ha^−1^) were selected for field testing. This set of lines had predicted values of 73.8–74.6 kg hl^−1^, 100–104 Julian days and 82.9–88.9 cm for TW, HD and PH, respectively (Supplementary Table S6, in green). Additionally, 20 lines with low predicted yield (3899–4056 kg ha^−1^) were included for comparison. These lines had predicted values for TW, HD and PH of 72.6–73.5 kg hl^−1^, 103–105 Julian days and 84.9–86.8 cm, respectively (Supplementary Table S6, in red).

### 3.4 QTL-by-environment

The historical phenotypic dataset containing YLD, TW, HD and PH measurements from many location-years (19 locations and 14 years, 2008–2021) (Figure 1 and Supplementary Table S4), along with information for presence/absence of 18 major effect QTL (Supplementary Table S3) for 1,172 for elite breeding lines, was used to study whether or not it was advantageous to harbor QTL under variable abiotic and biotic pressures across geographic space (Lowry et al., 2019). Using a linear mixed model, EMMs were calculated for six pairwise combinations of QTL (absent/present) and regions (1, 2 and 3) (Supplementary Table S7), and *p*-values (Supplementary Table S8) for nine pairwise comparisons across combinations. To consider genetic background bias, EMMs for YLD and *p*-values (*p* < 0.05) were also calculated based on origin of the breeding lines (Supplementary Tables S7, S8).

For each of the studied QTL (Supplementary Table S7), lines tested in region 1 had the highest EMMs of observed YLD followed by region 2, with region 3 demonstrating the lowest YLD potential. For each QTL, significant differences in YLD between regions 1 and 3 were most frequent. In most QTL-by-environment combinations, there were no significant differences in EMMs of YLD within regions when carrying or not carrying the major QTL (Supplementary Table S8). For test weight, region 2 showed the lowest EMM values (Supplementary Table S7); however, there were no significant differences with regions 1 and 3, nor within regions when comparing major effect QTL presence/absence (Supplementary Table S8). According to EMMs, heading date was later in region 1 (Supplementary Table S7) as expected, which was significantly different from regions 2 and 3 (Supplementary Table S8). Refer to Supplementary Tables S7, S8 for detailed information for each comparison of all 18 QTL and three testing mega-environments.

Five genes that are relevant for target population of environments across the southeastern US were more rigorously assessed individually for YLD trends within testing regions and by breeding program (e.g., region 1 observed data only included breeding lines developed and selected from a program located within region 1):

#### • Fhb1

**FIGURE 4:**
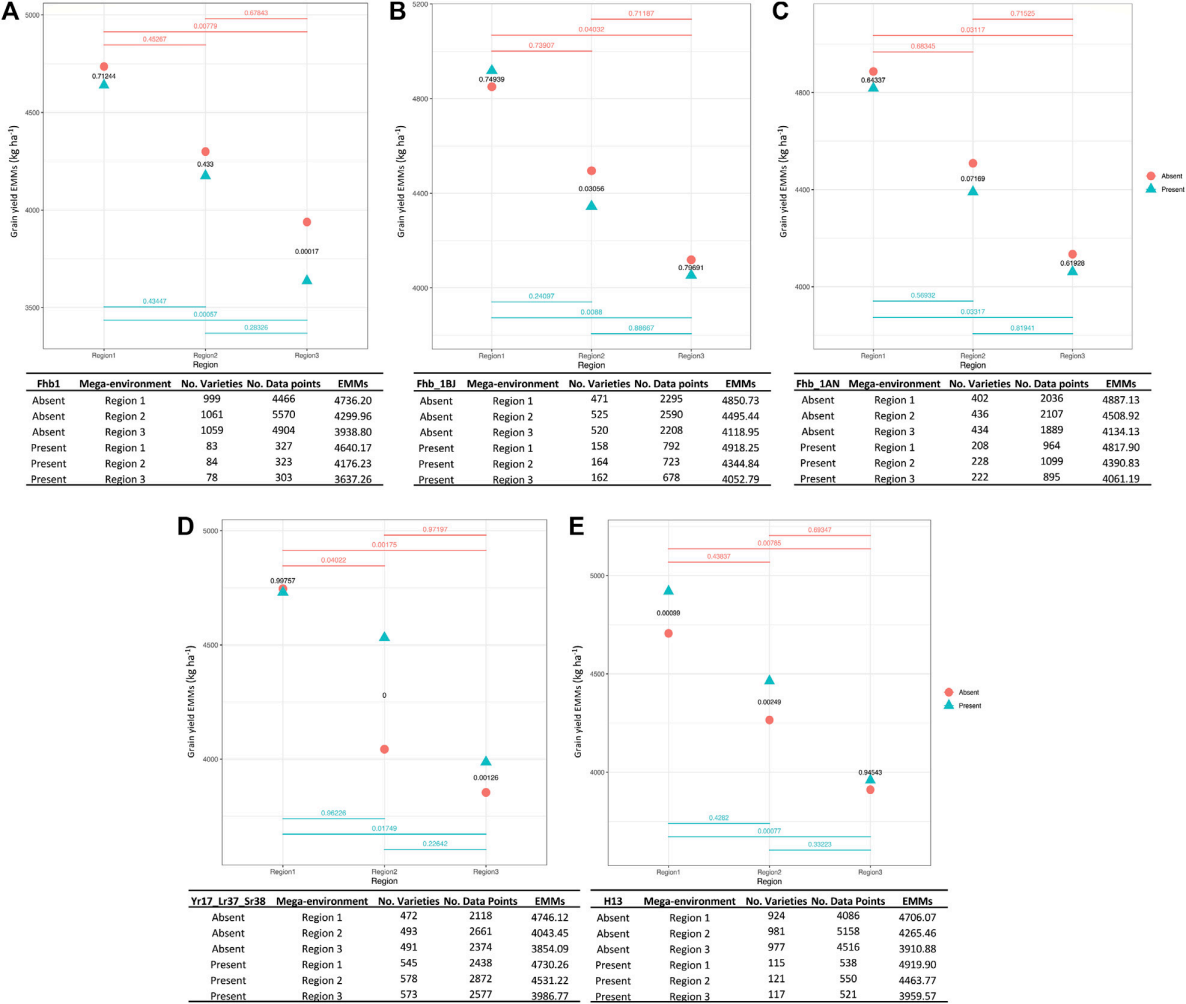
QTL-by-environment plots for YLD when five major genes are present or absent within three major testing regions (target population of environments or mega-environments). *Fusarium* head blight (FHB) (*F. graminearum*) resistance genes, Fhb1 **(A)**, Fhb_1B derived from ‘Jamestown’ cultivar **(B)**, Fhb_1A derived from ‘Neuse’ cultivar **(C)**, stripe rust (*P. striiformis*) resistance gene Yr17_Lr37_Sr38 **(D)** and hessian fly (*M. destructor*) resistance gene H13 **(E)**. Three regions in *x*-axis and EMMs calculated for YLD in *y*-axis. *p*-values are indicated for each pairwise comparison.

The FHB resistance gene Fhb1 (Yao et al., 1997), which first originated from ‘Sumai 3’, is located on chromosome 3BS had an overall frequency of 7.3% among 1,147 breeding lines. We found that wheat breeding lines harboring this gene had lower grain yield in all testing environments; however, this difference was only significant in the Gulf Coast (region 3) (Figure 4A).

#### • Fhb_1BJ

Fhb_1B (Wright 2014), an additional FHB resistance QTL derived from the cultivar ‘Jamestown’ (Griffey et al., 2010), had an overall frequency of 23.8% among 689 breeding lines. Lines carrying the resistance allele exhibited significantly lower YLD in region 2. Yields were similar for Fhb_1B carrying or non-carrying lines in regions 1 and 3 (Figure 4B).

#### • Fhb_1AN

This third FHB resistance QTL under study (Petersen et al., 2016) was derived from the cultivar ‘NC-Neuse’ (Murphy et al., 2004) and is located on chromosome 1A. The resistance allele at Fhb_1A exhibited a relatively high frequency of 34.3% among 664 breeding lines. Comparisons between lines with or without Fhb_1A mirrored Fhb_1B, where only region 2 exhibited a significantly lower YLD for lines possessing the resistance allele (Figure 4C).

#### • Yr17_Lr37_Sr38

The multi-functional rust resistance QTL is located in the 2N^V^S:2A translocation segment derived from *Aegilops ventricosa* (Gao et al., 2021). This QTL showed a consistently high introgression frequency of 53.9%, based on data from 1,072 breeding lines. The favorable allele for rust resistance had a consistent, positive effect on YLD (Figure 4D), especially in regions 2 and 3 where rust often threatens wheat production (Aboukhaddour et al., 2020).

#### • H13

This effective Hessian fly resistance gene was introgressed from *Aegilops tauschii* and is located on 6DS (Liu et al., 2005). H13 displayed an overall frequency of 11.0% among 1,104 breeding lines. Assessing its impact on productivity across the regions, the resistance allele at H13 had a significantly positive effect on YLD (Figure 4E).

When narrowing phenotypic data by only including breeding lines that originated within region, similar trends between presence/absence of these five major effect QTL and YLD were observed (Supplementary Figure S5). In other words, YLD trends largely held true to suggest genetic background was not impacting this analysis. A notable exception was lines that originated in region 1 and carried the Yr17_Lr37_Sr38 introgression segment yielded significantly greater than lines not harboring this QTL for rust resistance (Supplementary Figure S6).

## 4 Discussion

### 4.1 Application and benefits of genomic prediction in cultivar development programs

Integration of GS and molecular breeding technologies into the cultivar development pipeline has enabled established programs to accelerate the rate of genetic gain for complex traits and speed up the breeding process (Voss-Fels et al., 2019), while helping to minimize costs (Crossa et al., 2017). Implementation of GS into existing breeding programs that once fully relied on phenotypic selection comes with the challenge of restructuring the breeding pipeline to efficiently deploy genomics-enabled breeding (Merrick et al., 2022). Merrick et al. (2022) reviewed the specific aspects to consider that affect a given model’s predictive ability in GS including: 1) establishment of optimum TPs where size, structure and composition, and genetic relatedness to the target population impact accuracy (Isidro y Sánchez and Akdemir 2021); 2) genotyping and incorporation of major genes into the GS models; 3) speed breeding to reduce generation time and double haploids for accelerated fixation of traits; 4) leveraging phenotypic data by conducting multi-environment (multiple locations and years) trials and accounting for genotype-by-environment interaction of complex traits (e.g., yield); 5) incorporating multiple, high-heritable correlated traits to improve prediction accuracy for low heritable complex traits (Merrick et al., 2022); 6) incorporating new technologies to aid GS models, such us high-throughput phenotyping of secondary traits to select complex traits (Rutkoski et al., 2016); and 7) utilizing machine (Montesinos-López et al., 2018) or deep learning (Montesinos-López et al., 2021) for model building to increase statistical power.

The Clemson University winter wheat cultivar development program was recently reestablished in 2017 and served as a case study. New or reestablished breeding programs often have limited resources and must make difficult decisions on how to best adopt genomics-enabled breeding. Though challenging, these programs have a unique flexibility in deploying technology to inform critical decisions such as sourcing initial germplasm to establish the genetic foundation, determining crossing combinations for greatest population variance, and capturing genotype-by-environment interaction for a specific target population of environments. Existing historical genomic and phenotypic resources for many lines tested across locations and years could significantly benefit emerging or re-emerging breeding programs. These comprehensive datasets, previously shown to enhance prediction accuracy (Tomar et al., 2021; Zhao et al., 2021), were leveraged through the SunGrains multi-institutional collaborative program, which continues to successfully develop and release commercial cultivars for the southeastern US. This study utilized historical data generated and compiled by SunGrains to identify best practices for leveraging available genomic and phenotypic data to determine optimal genetic foundation for a specific target population of environments, and to incorporate robust GS models with high prediction accuracy. Here, grain yield data was used to cluster locations by correlated line performance (Boyles et al., 2019) into 22 groups, and optimization of TPs was implemented for each set with STPGA. Selecting TPs genetically related to new lines being evaluated should lead to an increase in prediction accuracy (Norman et al., 2018). In addition, it has been shown that accuracy in wheat increases with the increase of TP size, with 300 individuals (Isidro et al., 2015; Michel et al., 2017) or even greater (Sarinelli et al., 2019) being reported as the optimal number. As such, 400 individuals were selected for each TP in this winter wheat case study (based on unpublished tests).

When correlating GEBVs with observed phenotypic data, validation using 58 SC breeding lines demonstrated that using the combined TP (complete data from all regional trials and years) produced the highest prediction accuracy for grain yield (as high as *r* = 0.62), and outperformed predictions made with TPs with reduced data. The complete dataset not only included more high-quality data for predictions, but also TPs selected from a historical pool of lines tested in multiple years and geographic regions aids in capturing a broader range of environmental conditions when compared to newly generated, population-specific TPs. In this case study, it was apparent that the historical phenotypic dataset using all data (combined GAWN + SunWheat over 14 years) effectively captured environments that were representative of the collection of locations in 2021 where the 58 SC breeding lines were tested. Specifically, grain yield GEBVs for 40 of the 58 lines (69%) used for validation correctly grouped with observed data (Figure 3A). This result reinforces the utility and value of preserving and using historical data for building genomic selection models for new programs, as well as the importance of having strong regional alliances to share data across breeding programs. These collaborative networks enable genomics-enabled breeding to reach its theoretical potential for enhancing genetic gain (Spindel and McCouch 2016; Xu et al., 2020). A separate GS validation study leveraging historical winter wheat data reported a similar prediction accuracy of *r* = 0.64, which consisted of 483 lines grown over a 9-year period (Sarinelli et al., 2019). Meanwhile, lower accuracies (*r* = 0.28–0.50) were observed when using training data of 318 lines collected over 11 years at six locations in France (Storlie and Charmet 2013) and data from 254 lines tested in Mexico during 2010 (Poland J. et al., 2012). Although quality of phenotype data was high, and GS has the potential to improve grain yield, these results also imply that the complex nature of this trait with a moderate broad-sense heritability (*r* ^*2*^ = 0.56) is highly affected by genotype-by-environment interactions (Crossa et al., 2017).

### 4.2 Assessment of the presence/absence of major effect QTL on regional productivity

Grain yield remains the primary target trait for winter wheat improvement, but there are other agronomic, quality (Tsai et al., 2020; Sandhu et al., 2022) and resiliency traits that undergo intensive selection (Singh et al., 2016; Laidig et al., 2021; Langridge and Reynolds 2021). In this study, trends between grain yield and allele presence at major effect QTL were examined using existing PCR-based markers (Díaz et al., 2012; Guedira et al., 2016; Mason et al., 2018) and historical multiyear, multi-location phenotypic data. For this specific case study in southern SRW wheat, selection for broad adaptation is of interest as seed companies desire covering large market regions with fewer products. Thus, determining the best combination of major effect QTL would be a valuable selection tool to guide future breeding decisions. Broad adaptation for winter wheat in the southeastern US is elusive because there are myriad diseases and pests that threaten yield but often to various levels across the entire region. This study sought to provide evidence for the most appropriate combination of resistance QTL with high yield potential in absence of any biotic stress.

Several major effect QTL conferring resistance to FHB were examined because the primary threat from *F. graminearum* infection is reduced grain quality and deoxynivalenol (DON) toxin contamination, with FHB not known to severely hinder yield unless present at epidemic levels (Rod et al., 2020). Thus, there was interest in determining whether yield drag was observed from introgression of exotic (e.g., Fhb1) or native (‘Jamestown’ Fhb_1B and ‘Neuse’ Fhb_1A) resistance QTL and assessing how environment influenced the yield/QTL relationship. Although Fhb1 is widely used in many breeding programs, it was present at very low frequency (∼7%) within the SunGrains’ wheat lines, and genotypes harboring this gene exhibited lower yield regardless of testing region. Fhb1 is derived from an unadapted cultivar (Yao et al., 1997), and progenies using this source of resistance could inherit undesired agronomic traits due to linkage drag. Therefore, breeding lines harboring this QTL might be discarded by breeders in the field when looking and selecting for outstanding performance and adaptation. Marker-assisted backcrossing using adapted recurrent parents is a strategy to break the linkage and develop lines that combine the Fhb1 resistance gene with desired agronomic traits (Jin et al., 2013). Otherwise, use of native FHB resistance genes, present at higher frequency (Fhb_1B with ∼24% and Fhb_1A ∼34%) and without yield penalty, is highly recommended. For instance, one of the most productive and adapted SRW wheat lines in the southern US, ‘Hilliard’, harbors FHB resistance derived from ‘Jamestown’ (Griffey et al., 2020). Further opportunities to improve and provide durable FHB resistance is the pyramiding of native resistance genes with complimentary (or novel) QTL (Castro Aviles et al., 2020).

The recent study by Gao et al. (2021) found a positive yield effect of Yr17_Lr37_Sr38 in the US Great Plains and across an international performance trial led by the International Maize and Wheat Improvement Centre (CIMMYT). Indeed, this same trend was observed in SRW wheat adapted to the southeastern US, irrespective of region, where lines that possessed the introgression segment from *A. ventricosa* exhibited significantly higher mean yields than lines not carrying this introgression. As such, it was not surprising to observe that approximately 50% of breeding lines in the study carried Yr17_Lr37_Sr38. Fixing this QTL in a breeding program would be suggested, given its multi-purpose rust resistance benefit and purported linkage to favorable yield gene(s). For H13, the QTL that confers strong resistance to the local biotype L Hessian fly, was present at much lower frequency (11%). Because of the tendency of Hessian fly biotype L to be more frequent and impactful along the Atlantic Coastal Plain (regions 1 and 2, Figure 1), it was not surprising to see that lines harboring the resistance allele at H13 had higher yield than non-H13 lines in these regions, especially given that Hessian fly is a yield-threatening pest. In region 3, where Hessian fly biotype L is less common, there were no yield differences between lines with or without H13 (Ratcliffe et al., 1994; Ratcliffe et al., 2000; Onstad and Knolhoff 2014).

## Conclusion

For most major food crops, there are extensive resources available, including in the public domain, that can be leveraged to rapidly scale new or reestablished breeding programs that do not have direct access to valuable germplasm, data, or selection tools at program inception. This study examined the reestablished soft red winter wheat breeding program at Clemson University to establish processes for integrating available resources to accelerate the time from program inception to cultivar release. These steps included 1) utilizing a combination of historical phenotype data and genome-wide SNP markers to build a reliable GS model for predicting best lines for a target population of environments, and 2) identifying major effect QTL using existing PCR-based marker reports that were favorable, within the context of region and biotic pressure. This study highlights the importance of cooperative efforts between breeding programs that share a target population of environments to not only perform extensive multi-environment field trials but also to compile genotypic and phenotypic datasets that are key to enhancing genetic gain through robust genomic prediction models.

## Data Availability

Genomic and phenotypic SunGrains datasets are not readily available. Requests to access these data should be directed to the corresponding author.

## Footnotes

2
https://www.mapcustomizer.com/

## Abbreviations

BLUPs, Best linear unbiased predictors; CV, Cross-validation; EMMs, Estimated marginal means; FHB, *Fusarium* head blight; GAWN, Gulf Atlantic wheat nursery; GBLUP, Genomic best linear unbiased prediction; GBS, Genotyping by sequencing; GE, Genotype-by-environment; GEBVs, Genomic estimated breeding values; GGE, Genotype plus genotype-by-environment; GS, Genomic selection; HD, Heading date; MAF, Minor allele frequency; PCA, Principal component analysis; PH, Plant height; QE, QTL-by-environment; R-gene, Resistance Gene; SNP, Single nucleotide polymorphism; SPE, SunGrains preliminary early nursery; SPL, SunGrains preliminary late nursery; SRW, Soft red winter wheat; STPGA, Selection of training population with a genetics algorithm; SunGrains, Southeastern University small GRAINS cooperative^1^; SunWheat, SunGrains advanced wheat nursery; TP, Training population; TW, Test weight; VCF, Variant call format; YLD, Grain yield.
