# Supplementary material for: Utilizing genomics and historical data to optimize gene pools for new breeding programs: A case study in winter wheat
Source: Front Genet. 2022 Oct 7;13:964684. doi: 10.3389/fgene.2022.964684 (PMC9585219; doi:10.3389/fgene.2022.964684)
Supplement: Supplementary file 1 [file DataSheet1.PDF]

Utilizing genomics and historical data to optimize gene pools for new breeding programs: A case study in winter wheat

Carolina Ballén-Taborda, Jeanette Lyerly, Jared Smith, Kimberly Howell, Gina Brown-Guedira, Md Ali Babar, Stephen A. Harrison, Richard E. Mason, Mohamed Mergoum, J. Paul Murphy, Russell Sutton, Carl A. Griffey, Richard E. Boyles

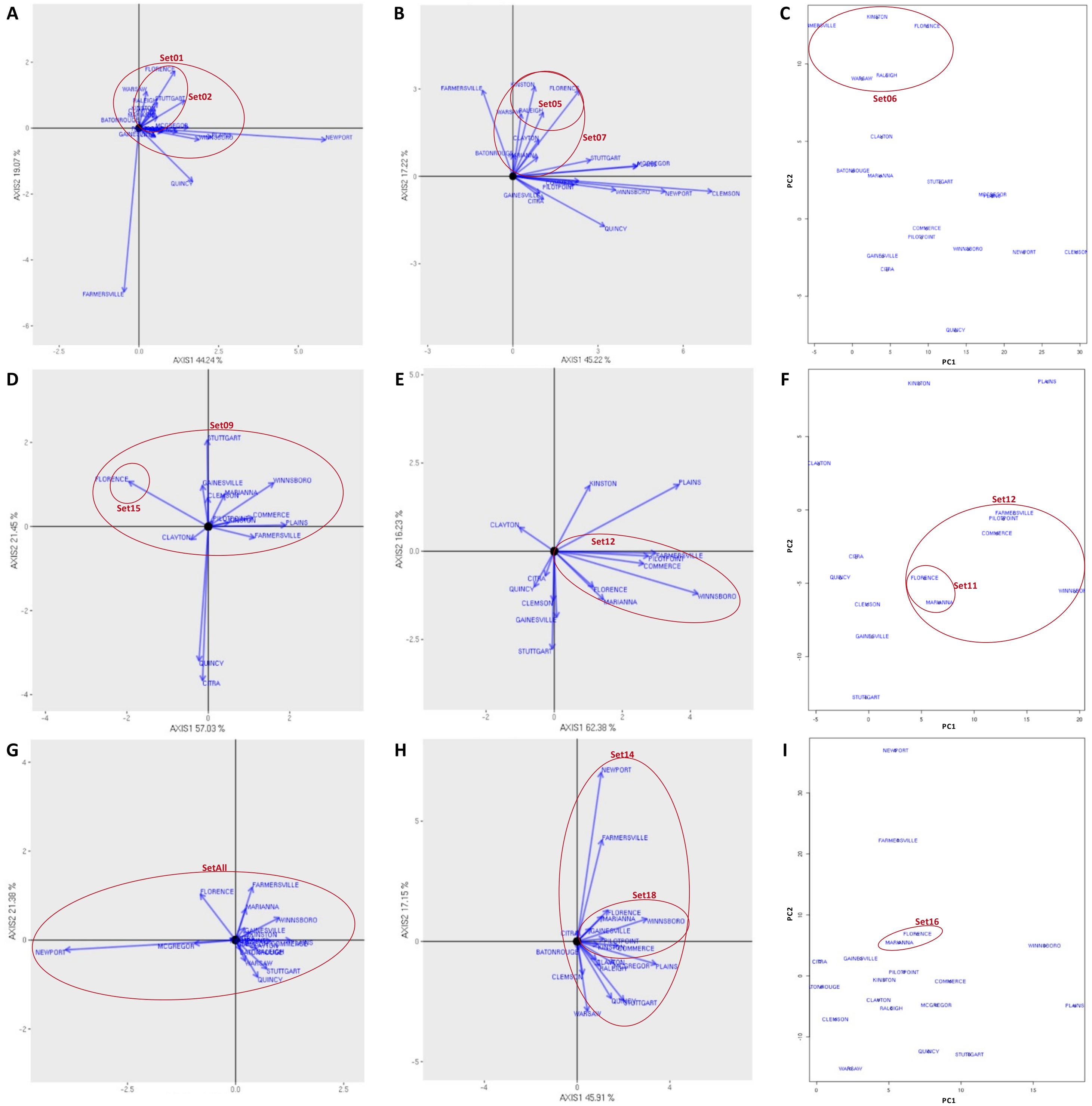

Figure S1: GGE biplots showing the relationship among environments and principal component (PCs) plots were calculated to select 22 subsets of locations. Using all data collected in GAWN nursery, biplot **(A)** was calculated to select Set01-Set02 subgroups, and biplot **(B)** and PC plot **(C)** displayed when removing Newport2020 and Florence2020 environments to select Set03-Set08 subgroups. Using all data collected in SunWheat nursery, biplot **(D)** was estimated by to select Set09-Set10 subgroups, and biplot **(E)** and PC plot **(F)** were displayed when removing Plains2014 and Florence2020 environments to select Set11-Set13 subgroups. Using all combined data collected in GAWN and SunWheat nurseries, biplot **(G)** was calculated to select Set15 and SetAll subgroups, and biplot **(H)** and PC plot **(I)** were displayed when removing Newport2020 and/or Florence2020 environments to select Set14, Set16-Set22 subgroups (Table S1). Examples of subset of locations are marked in red.

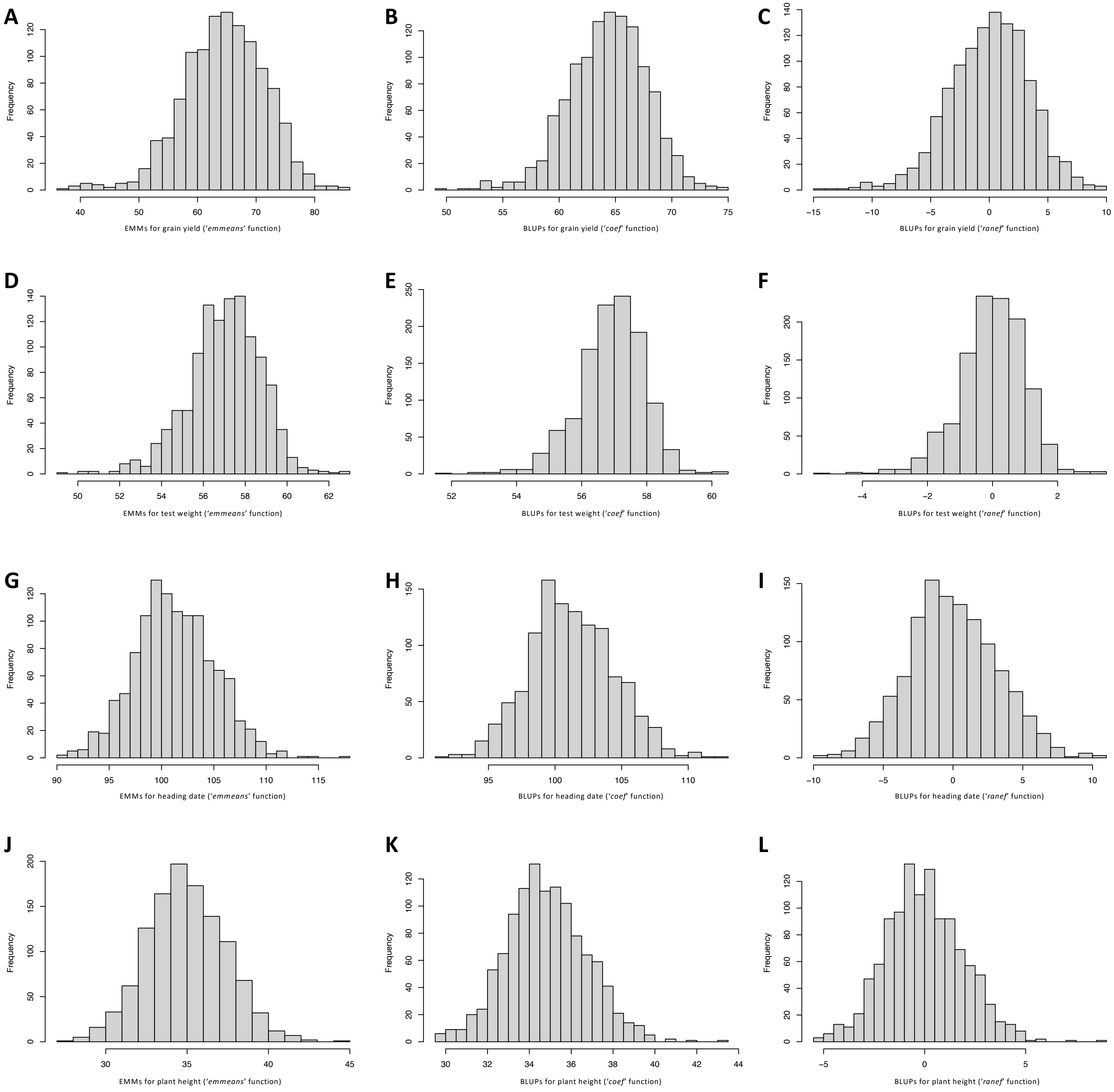

Figure S2: Frequency distribution of EMMs (*'emmeans'* function) and BLUPs (*'coef'* and *'ranef'* functions) calculated using the full phenotypic dataset (SetAll) for YLD **(A, B, C)**, TW **(D, E, F)**, HD **(G, H, I)** and PH **(J, K, L)**. Estimated values in x-axis and frequency of breeding lines in y-axis. Similar trend was observed for all subset of locations (data not shown).

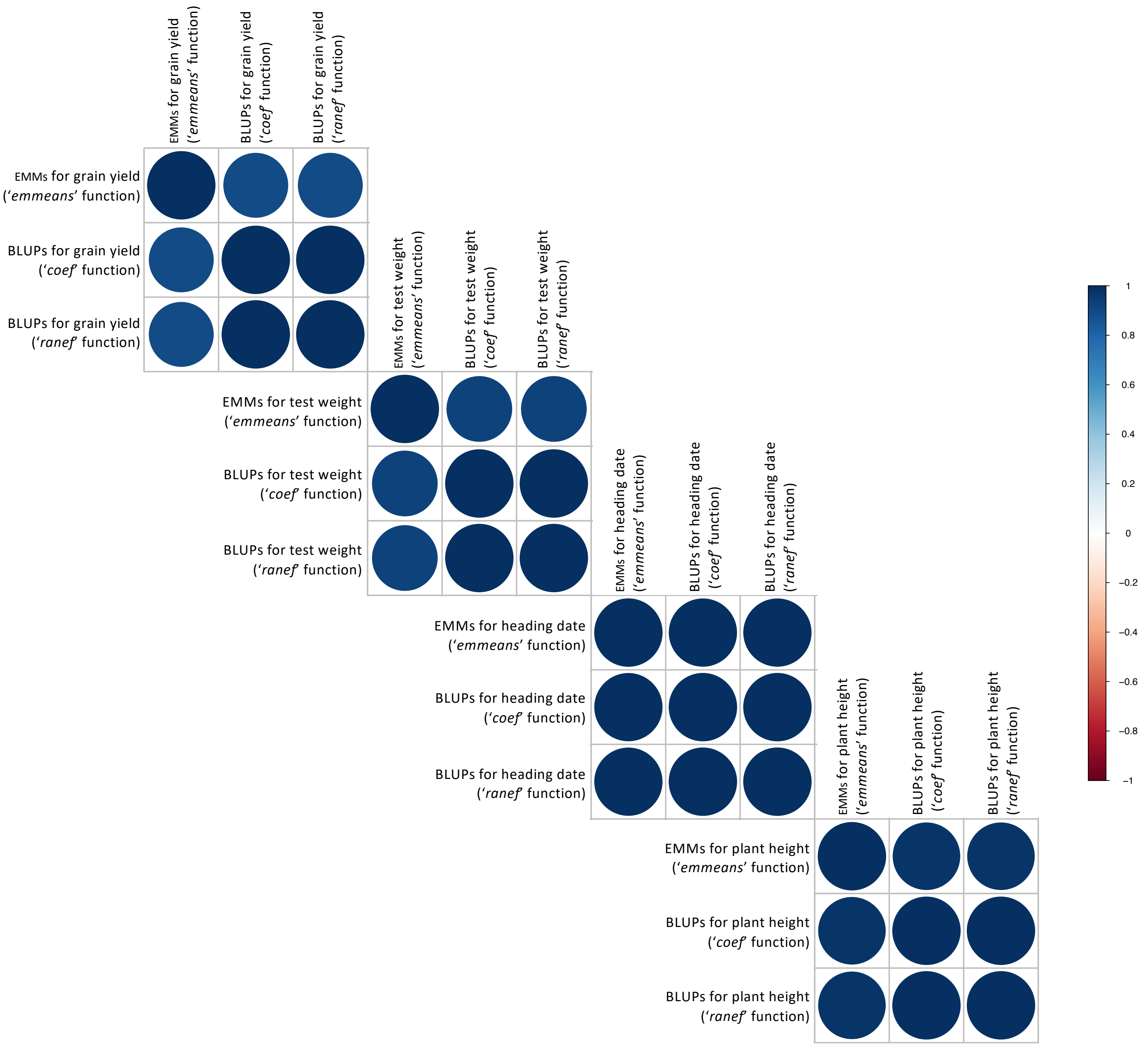

Figure S3: Pearson's pairwise correlation plot between EMMs (*'emmeans'* function) and BLUPs (*'coef'* and *'ranef'* functions) calculated using the full phenotypic dataset (SetAll) for YLD, TW, HD and PH. Dark blue and bigger circles indicating strong positive association between estimated values. Similar trend was observed for all subset of locations (data not shown).

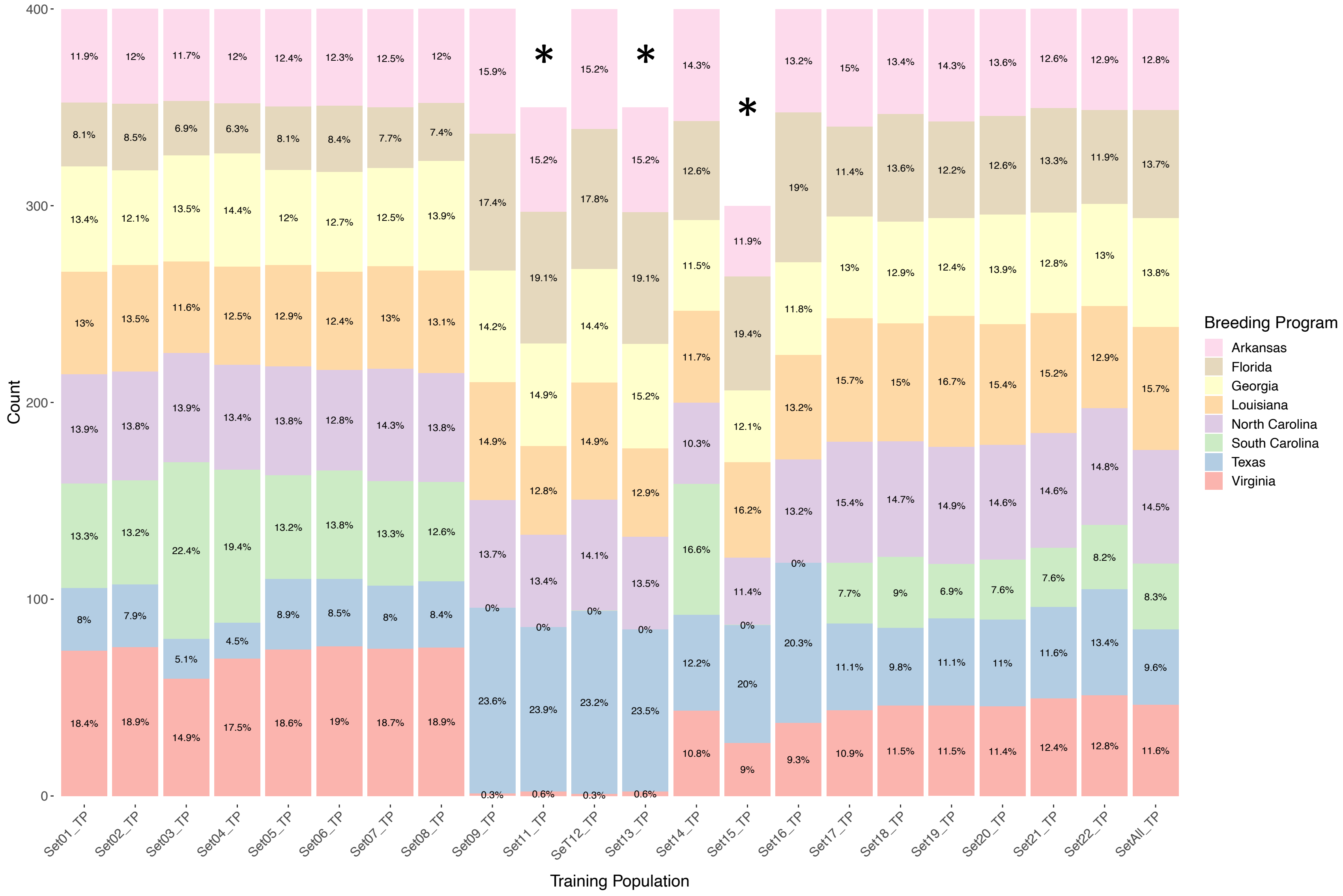

Figure S4: Stacked barplot showing representation of breeding programs within training populations (TPs) selected by *'STPGA'* R package. Normalized percentage (%) of total lines by program is presented inside colored sections of each bar. TPs in x-axis and normalized counts of breeding lines in y-axis. On top of three bars, "*" indicate TPs with fewer lines selected due to lower entries available. For more details refer to Table S2.

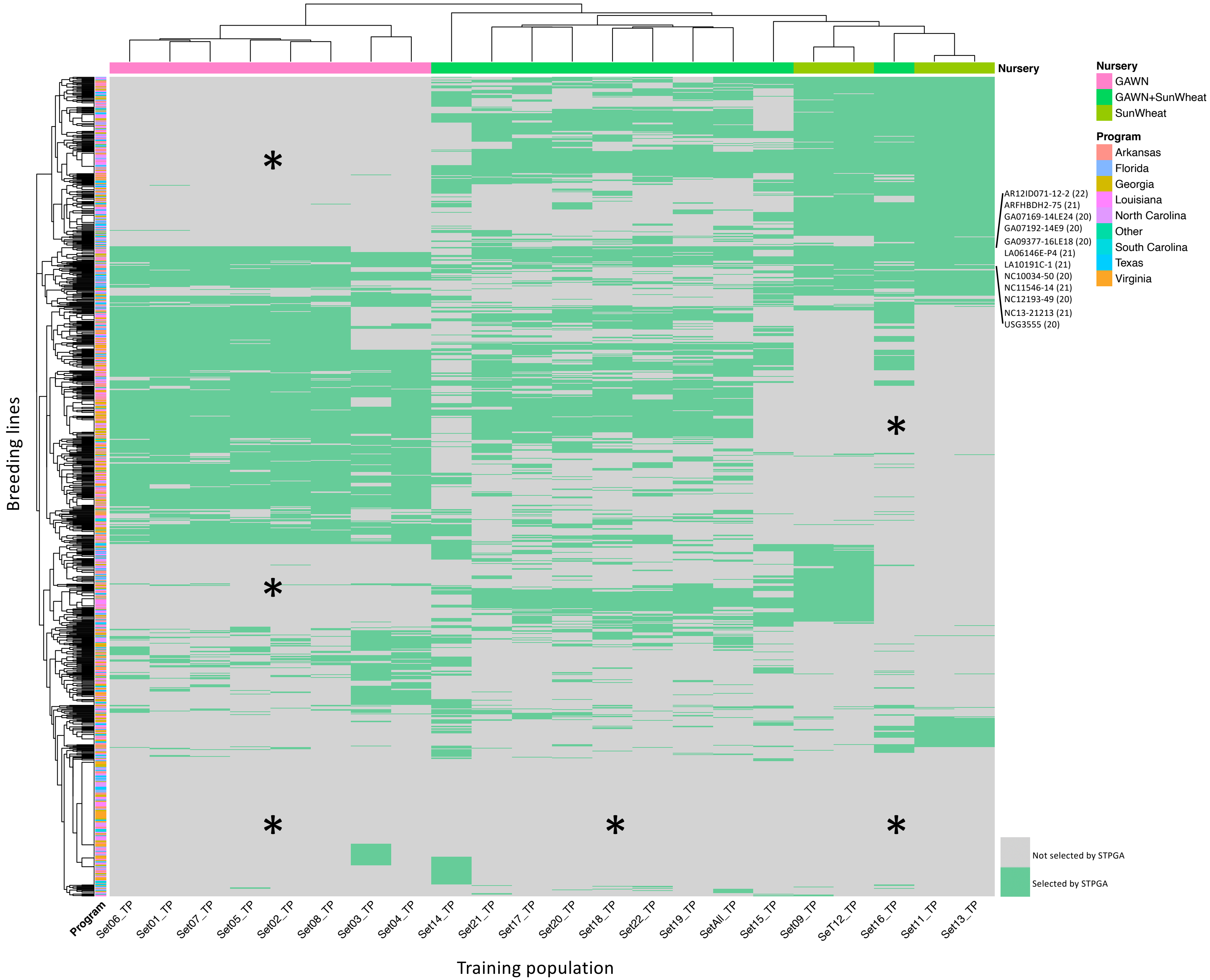

Figure S5: Heatmap displaying hierarchical clustering of 22 TPs (x-axis, cluster and nursery information presented on top of the figure) selected by *'STPGA'* R package and SunGrains' elite lines (y-axis, cluster of entries and breeding program shown to the left). Breeding lines that were selected by *'STPGA'* in green and not selected lines in gray. Twelve most present lines across TPs are listed to the right of the figure, with the number of TPs in which those were included in parenthesis. Gray blocks indicated by "*" represent breeding lines that were not available for selection of TP.

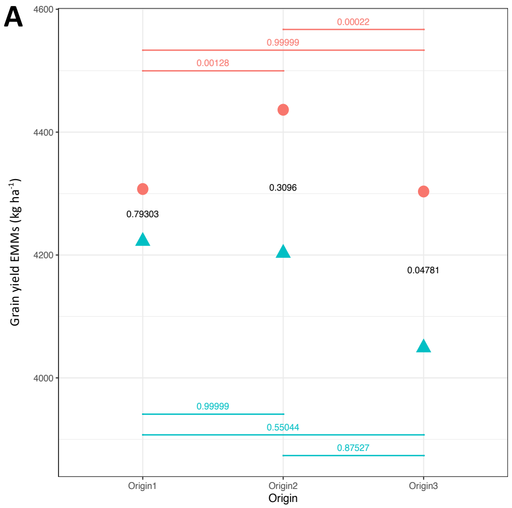

Fhb1	Origin	No. Varieties	No. Data points	EMMs
Absent	Origin 1	338	4965	4307.39
Absent	Origin 2	278	4185	4436.39
Absent	Origin 3	447	5790	4303.37
Present	Origin 1	46	533	4222.44
Present	Origin 2	14	155	4203.02
Present	Origin 3	24	265	4049.11

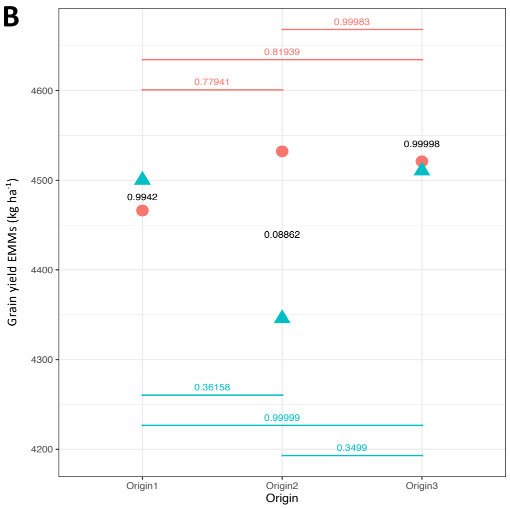

Fhb_1BJ	Origin	No. Varieties	No. Data points	EMMs
Absent	Origin 1	122	1717	4466.23
Absent	Origin 2	141	2060	4532.18
Absent	Origin 3	262	3316	4521.02
Present	Origin 1	65	958	4500.23
Present	Origin 2	48	569	4345.82
Present	Origin 3	51	666	4510.53

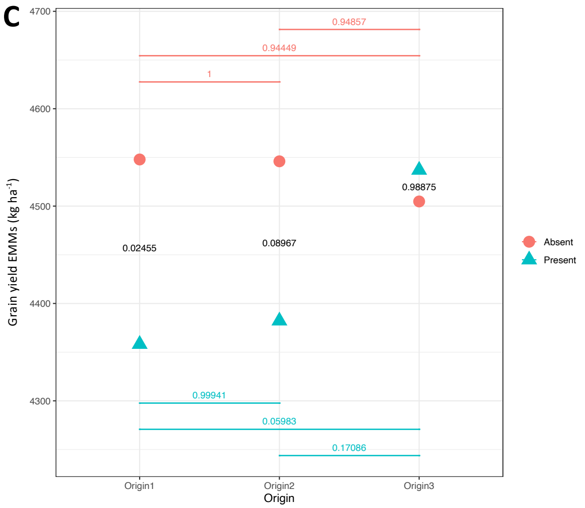

Fhb_1AN	Origin	No. Varieties	No. Data points	EMMs
Absent	Origin 1	116	1729	4547.88
Absent	Origin 2	126	1898	4546.00
Absent	Origin 3	194	2405	4504.76
Present	Origin 1	68	907	4358.15
Present	Origin 2	65	813	4382.15
Present	Origin 3	95	1238	4536.96

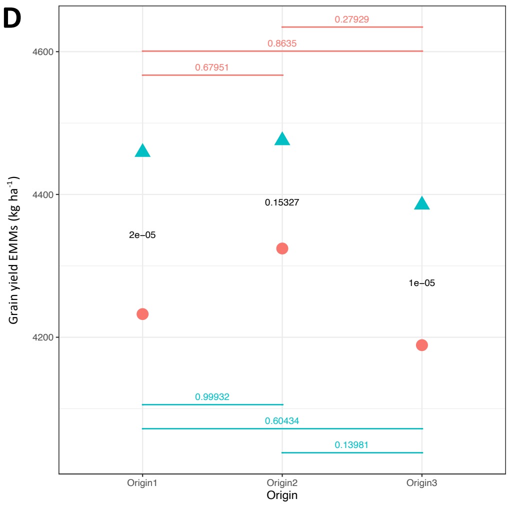

Yr17_Lr37_Sr38	Origin	No. Varieties	No. Data points	EMMs
Absent	Origin 1	256	3938	4232.45
Absent	Origin 2	50	744	4324.31
Absent	Origin 3	188	2471	4188.96
Present	Origin 1	106	1379	4459.09
Present	Origin 2	221	3310	4475.50
Present	Origin 3	251	3198	4385.40

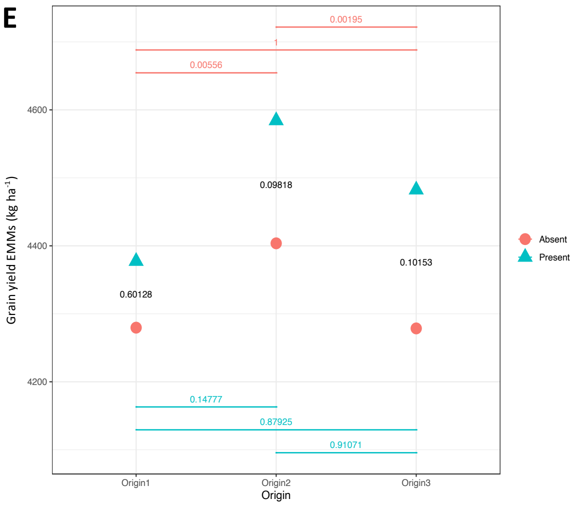

H13	Origin	No. Varieties	No. Data points	EMMs
Absent	Origin 1	318	4678	4279.63
Absent	Origin 2	237	3500	4403.66
Absent	Origin 3	428	5582	4278.51
Present	Origin 1	52	687	4377.32
Present	Origin 2	38	597	4584.22
Present	Origin 3	31	325	4482.37

Figure S6: QTL-by-environment plots for YLD when five major genes are present or absent within three major locations of origin (where breeding lines were developed). Fusarium head blight (FHB) (*F. graminearum*) resistance genes, Fhb1 (**A**), Fhb_1B derived from 'Jamestown' cultivar (**B**), Fhb_1A derived from 'Neuse' cultivar (**C**), stripe rust (*P. striiformis*) resistance gene Yr17_Lr37_Sr38 (**D**) and hessian fly (*M. destructor*) resistance gene H13 (**E**). Three regions in x-axis and EMMs calculated for YLD in y-axis. P-values are indicated for each pairwise comparison.
